# The brain-structural correlates of mathematical expertise

**DOI:** 10.1016/j.cortex.2018.10.009

**Published:** 2019-05

**Authors:** Tudor Popescu, Elie Sader, Marie Schaer, Adam Thomas, Devin B. Terhune, Ann Dowker, Rogier B. Mars, Roi Cohen Kadosh

**Affiliations:** a Department of Experimental Psychology, University of Oxford, Oxford, UK; b Wellcome Integrative Neuroscience Centre, University of Oxford, Oxford, UK; c Department of Psychiatry & Behavioral Sciences, Stanford University, Palo Alto, CA, USA; d FMRIF, NIMH, NIH, Bethesda, MD, USA; e Department of Psychology, Goldsmiths, University of London, London, UK; f Donders Institute for Brain, Cognition and Behaviour, Nijmegen, Netherlands

**Keywords:** Mathematics, Expertise, Numerical cognition, Grey matter, White matter

## Abstract

Studies in several domains of expertise have established that experience-dependent plasticity brings about both functional and anatomical changes. However, little is known about how such changes come to shape the brain in the case of expertise acquired by professional mathematicians. Here, we aimed to identify cognitive and brain-structural (grey and white matter) characteristics of mathematicians as compared to non-mathematicians. Mathematicians and non-mathematician academics from the University of Oxford underwent structural and diffusion MRI scans, and were tested on a cognitive battery assessing working memory, attention, IQ, numerical and social skills. At the behavioural level, mathematical expertise was associated with better performance in domain-general and domain-specific dimensions. At the grey matter level, in a whole-brain analysis, behavioural performance correlated with grey matter density in left superior frontal gyrus – positively for mathematicians but negatively for non-mathematicians; in a region of interest analysis, we found in mathematicians higher grey matter density in the right superior parietal lobule, but lower grey matter density in the right intraparietal sulcus and in the left inferior frontal gyrus. In terms of white matter, there were no significant group differences in fractional anisotropy or mean diffusivity. These results reveal new insights into the relationship between mathematical expertise and grey matter metrics in brain regions previously implicated in numerical cognition, as well as in regions that have so far received less attention in this field. Further studies, based on longitudinal designs and cognitive training, could examine the conjecture that such cross-sectional findings arise from a bidirectional link between experience and structural brain changes that is itself subject to change across the lifespan.

## 1 Introduction

With the ever-growing presence of numerical information in our environment, those endowed with good abilities of understanding, evaluating and manipulating numbers and mathematical information have a greater chance to do well in life (Estrada et al., 2004, Parsons and Bynner, 2005). Furthermore, it has been suggested that the contribution of numeracy to different indices such as employment, education, and physical and mental wellbeing is even more substantial than that of literacy (Beddington et al., 2008, Bynner and Parsons, 1997). Surprisingly, little is known about the link between brain structure and mathematical excellence, as more emphasis is given in the literature to the neural correlates of mathematical deficits than to those of mathematical expertise. However, improved understanding of *both* of these aspects is necessary if we are to attain a more holistic knowledge of mathematical cognition and its neural correlates – with implications for psychology, education, and neuroscience.

Several neuroimaging studies with healthy volunteers have consistently identified a number of brain regions that are involved in the performance of arithmetic tasks (Andres et al., 2011, Grabner et al., 2009, Kong et al., 2005, Rivera et al., 2005). The intraparietal sulcus (IPS) is the region that is most often reported in tasks that involve basic numerical processing (Arsalidou and Taylor, 2011, Cohen Kadosh et al., 2008, Kaufmann et al., 2011, Knops and Willmes, 2014). This includes tasks involving both symbolic and non-symbolic numbers (Harvey et al., 2013, Piazza et al., 2004), linked to mathematical achievements (De Smedt et al., 2013, Schneider et al., 2017). However, the network of areas activated during basic and more advanced number processing is much wider, and is distributed across the parietal lobe (Delazer et al., 2003, Grabner et al., 2009, Wu et al., 2009), as well as involving the prefrontal cortex (Kaufmann et al., 2011, Nieder and Dehaene, 2009), and other cortical and subcortical regions (Dehaene and Cohen, 1997, Hittmair-Delazer et al., 1994, Menon, 2015, Qin et al., 2014). In addition, the contribution of frontal and parietal cortices to numerical processing varies across development and with experience, with parietal functions gradually increasing their involvement (Ansari et al., 2005, Cantlon et al., 2006, Rivera et al., 2005), which might be associated with less effortful and more automatic processing (Cohen Kadosh & Walsh, 2009).

Of special interest is the study of the function and structure of these cortices in experts. A recent study found increased activation across a network of bilateral frontal, intraparietal and ventrolateral temporal regions, as mathematician academics evaluated statements from several domains of mathematics (Amalric & Dehaene, 2016); such activation was not found in a group of non-mathematician academics. On the other hand, at the structural level, another study investigated differences between mathematicians and non-mathematicians (Aydin et al., 2007) – the only such study to date, to our knowledge. Aydin et al. found higher grey matter density (GMD) in the left inferior frontal gyrus (IFG) and bilateral inferior parietal lobule (IPL) for mathematicians versus non-mathematicians, which was correlated with the number of years spent as a mathematician. However, one of the critical limitations in that study was that the level of mathematical education was not controlled for in the two groups, and subjects were not cognitively tested, which did not enable the authors to link their brain-structural results to behavioural performance. Nevertheless, the results from both studies mentioned above show overlap in the neural correlates of mathematical expertise, as reflected in the direct contrasts between mathematicians and non-mathematicians. These areas included the left inferior/middle frontal gyrus [Brodmann area (BA) 46] and the right intraparietal sulcus (BA 7). However, the link between structural metrics of these areas and the cognitive abilities associated with the individuals was lacking in both studies – and motivates the present one.

A brain-structural basis to individual differences and expertise in mathematics has been suggested not only with regards to the cortical networks involved, but also to the various white matter tracts connecting these networks' nodes (for a review, see Moeller, Willmes, & Klein, 2015), with fractional anisotropy (FA) being a common metric to quantify their microstructural integrity. For instance, in adults, Matejko, Price, Mazzocco, and Ansari (2013) found performance in an arithmetic test to correlate with FA in the left superior longitudinal fasciculus (SLF), superior corona radiata (SCR) and the cortico-spinal tract; while van Eimeren et al. (2010) found that FA in a central segment of the left SCR correlated with the blood-oxygen-level dependent (BOLD) response in the left angular gyrus, during a retrieval-prone calculation task. Also, Navas-Sánchez et al. (2014) found, in math-gifted adolescents as compared to controls, higher FA in the corpus callosum and in tracts including the thalamic radiations and the inferior longitudinal fasciculi. Finally, Tsang, Dougherty, Deutsch, Wandell, and Ben-Shachar (2009) found that mean FA in the left anterior portion of the SLF (aSLF) – as delineated using tractography – of healthy adolescents correlates with their performance in a mental approximate calculation task, even after co-varying out age and scores on other more general cognitive tasks.

At the cognitive level, several studies have shown exceptional mathematical ability to be associated with exceptional performance in domain-general cognitive processes such as working memory, long-term memory and visual imagery. These associations arise from both historic-anecdotal evidence (e.g., Hadamard, 1949, Scripture, 1891) as well as case-studies of arithmetical prodigies (e.g., Fehr et al., 2010, Pesenti et al., 2001). The role of supporting (auxiliary) functions – such as long-term memory, working memory, attention and mental imagery – in numerical competence is evident from a large body of literature that includes studies linking individual differences in those domain-general functions to numerical performance (Bull et al., 2008, Dumontheil and Klingberg, 2012, O'Boyle, 2008, Pesenti et al., 2001, Thompson et al., 2013, van Dijck and Fias, 2011), cognitive training studies (Looi et al., 2016, Nemmi et al., 2016, Takeuchi et al., 2011), as well as studies reporting impairment in these functions that are concomitant with numerical deficits (Passolunghi and Siegel, 2004, Wilson and Swanson, 2001). Based on these results we included in our testing battery both domain-specific and domain-general tasks.

Motivated by the literature reviewed in this section, our aim in the current study was to examine whether and how mathematicians are different from non-mathematician controls at the neural and cognitive level. To do so we used voxel-based morphology for grey matter and diffusion measures for white matter, in conjunction with an extensive cognitive assessment. We predicted cortical differences between mathematicians and non-mathematicians in prefrontal and parietal cortices, in particular the inferior frontal gyrus, the intraparietal sulcus and the inferior/superior parietal lobule – with no specific prediction regarding which group would show increased GMD values; and differences in the integrity of the white matter tracts radiating from these structures, in particular increased FA in the SLF of mathematicians. We expected both domain-specific and domain-general behavioural differences; namely, we expected mathematicians to obtain higher scores in numerical, working-memory and visuo-spatial tasks; but lower scores in social and language-related tasks. Moreover, we examined whether any group brain differences have behavioural relevance by computing correlations between the different brain indices and an aggregated index of behavioural performance.

## 2 Methods

### 2.1 Subjects

Thirty-eight subjects (all right handed; 10 females) were recruited from the University of Oxford. All were academics either at doctoral or post-doctoral level. The mathematicians group consisted of 19 subjects (5 females, μ = 25.7, σ = 1.6, range = 23.2 to 32.9) working in various areas of mathematics (such as Algebra, Logic and Number Theory) at the Mathematical Institute of the University of Oxford. The non-mathematicians control group^1^ also consisted of 19 subjects (14 males, μ = 26.3, σ = 2.4, range = 24.2 to 30.4) from the field of humanities (Departments of English, Modern Languages, Classics and History). Subjects were reimbursed at £10 per hour. The research has been approved by the Berkshire Research Ethics Committee.

### 2.2 Behavioural tasks

We used a battery of tests to map possible group differences in several cognitive abilities including intelligence (IQ test; Wechsler, 1999), working memory (digit span; Wechsler, 1997), attention (Attentional Networks Test, ANT; Fan, McCandliss, Sommer, Raz, & Posner, 2002), mental imagery (Mental Rotation Task, MRT; Peters et al., 1995), various numerical and logical skills (e.g., numerical Stroop; Henik & Tzelgov, 1982; number line; Siegler & Opfer, 2003), and various verbal reasoning and social skills (e.g., the autism spectrum quotient, ASQ; Baron-Cohen, Wheelwright, Skinner, Martin, & Clubley, 2001). These tests are summarised in Table S1 of the Supplementary Materials. Additionally, all subjects completed a questionnaire asking them to rate (on a scale from 1 to 10) the degree to which they use the following different modalities (strategies) when solving arithmetical problems: visuo-spatial (trying to visualise the numbers or equations), outer verbalisation, inner verbalisation or kinaesthetic (finger counting). Since the proportion of native English speakers was different in the two groups [10 out of 19 mathematicians and 17 out of 19 non-mathematicians; χ^2^(1) = 6.27, *p* < .05], we included this factor as a covariate when testing for group differences in tasks having a strong linguistic component (verbal IQ and verbal reasoning).

### 2.3 MRI acquisition

All subjects were scanned using the Siemens 3T scanner at the Oxford Centre for Functional Imaging of the Brain (FMRIB). T1-weighted anatomical images were acquired in the sagittal plane using an MPRAG sequence (TR = 15.7 msec; TE = 4.6 msec; ﬂip angle = 8, inversion time = 900 msec, voxel size = 1 × 1 × 1 mm). In addition, whole brain diffusion weighted volumes were acquired [60 directions; b = 1000 sec/mm^−2^; 65 slices; voxel size 2 × 2 × 2 mm; repetition time (TR) = 9.6 sec; echo time (TE) = 87 msec] plus eight volumes without diffusion weighting (b = 0 sec/mm^−2^).

### 2.4 Analyses

All brain analyses were conducted in version 5.0.5 of the FMRIB Software Library (FSL; Oxford Centre for Functional MRI of the Brain, University of Oxford, UK).

#### 2.4.1 Grey matter

In order to compare GMD across groups, we used Voxel-Based Morphometry (VBM), which compares local grey matter tissue volumes at the voxel level. For the whole-brain level analysis, we followed the optimised VBM procedure (Good et al., 2001). Namely, all structural images were brain-extracted, segmented according to tissue type (white matter, grey matter, cerebrospinal fluid), aligned to MNI space, nonlinearly registered and modulated (divided by the Jacobian of the warp field used for registration). The images thus processed were smoothed using a Gaussian kernel with σ = 4 mm (corresponding to a full-width at half-maximum of 9.4 mm), and voxel-wise general linear model (GLM) was applied using FSL's permutation-based non-parametric testing, with corrections for multiple comparisons across space. For group comparisons, group was entered into the design matrix as a categorical predictor; gender and age were entered as categorical and continuous (respectively) predictors of no interest (nuisance variable). For brain-behaviour correlations, behavioural score was additionally entered as a continuous predictor; the design was also repeated within each group separately, after eliminating group as predictor.

As an additional analysis to the whole-brain level, we selected a priori regions of interest (ROIs) known to be involved in the mathematical brain network, as per the literature reviewed above. Centre coordinates for the bilateral intraparietal sulcus (IPS), superior parietal lobule (SPL), and inferior frontal gyrus (IFG) were based on a recent meta-analysis of functional studies in the field of numerical cognition (Arsalidou & Taylor, 2011) and on the Jülich histological atlas (Eickhoff, Heim, Zilles, & Amunts, 2006) as implemented in FSL. These centre coordinates were: left IPS: x = −53, y = −32, z = 33, BA 40; right IPS: x = 40, y = −40, z = 43, BA 40; left SPL: x = −26, y = −60, z = 46, BA 7; right SPL: x = 30, y = −62, z = 44, BA 7; left IFG: x = −42, y = 4, z = 30, BA 9; right IFG: x = 46, y = 10, z = 28, BA 9. Following previous procedures (de Oliveira-Souza et al., 2008, Landgrebe et al., 2009), a sphere of radius 8 mm (volume 2,144 mm^3^) was built around each centre, in order to create individual ROI masks from which the average GMD was then extracted using FSL's *fslstats* tool. Statistical analyses were subsequently performed on these extracted values, using external software (STATISTICA, StatSoft Inc.; and MATLAB, MathWorks Inc.).

#### 2.4.2 White matter

Distortions in the DTI images due to subject motion or Eddy currents were corrected by applying affine alignment of each image to the non-diffusion image (b_0_), using FMRIB's diffusion toolbox (FSL, version 5.0.5, www.fmrib.ox.ac.uk/fsl; Smith et al., 2004). Diffusion anisotropy was quantitatively measured by FA (fractional anisotropy) (Basser & Pierpaoli, 1996). All eigenvalues (λ_1_, λ_2_, λ_3_) and FA maps were calculated using FSL's DTIFit tool. After aligning each subject's FA map to standard space (MNI152), tract-based spatial statistics (TBSS) were carried out in FSL for all FA images, using the procedure described in (Smith et al., 2006). In short, a mean FA image and a “skeletonised” FA image is created from all subjects' aligned FA maps, and each subject's FA map is then projected onto the skeleton. Finally, voxel-wise statistics are made across subjects for all skeletonised FA images. As with the grey matter analyses, we included age and gender as covariates.

For the ROI analysis, the masks included the white matter regions that underlie the ROIs chosen for the grey matter analysis. The masks were defined as spheres of a larger radius (12 mm), in order for the sphere to overlap the FA skeleton. We extracted voxel intensities (which in this case represented FA values) from the skeletonised FA images.

Mean diffusivity (MD) was also computed from the diffusion data. MD is defined as the average of the three eigenvalues, and represents a measure of the average diffusion in a voxel that is complementary to that provided by FA. Higher values of MD are associated with decreased barriers to water diffusion in white matter. Whereas FA indicates the relative distance between the largest eigenvalue and the others, MD provides an average of the three eigenvalues and thus represents the overall amount of diffusion. The two measures have been shown to relate to different aspects of the underlying anatomy and can be differentially related to behavioural variables (Johansen-Berg & Rushworth, 2009).

In addition to voxel-wise FA and MD values, we also used probabilistic tractography (Behrens et al., 2003, Behrens et al., 2003), a technique for identifying fibre pathways from diffusion-weighted imaging, to reconstruct the anatomical networks linking the brain regions that are relevant to mathematics. We first created a mask of the bilateral aSLF II – a pathway connecting parietal and frontal cortex – by identifying it in the JHU White-Matter Tractography Atlas and setting a population threshold of 20%; that meant that, in order for a voxel to be considered as part of the tract, it had to be identified as the aSLF in at least 20% of the individual brains that make up the probabilistic atlas. From this tract, we then kept the same seven coronal planes identified in (Tsang et al., 2009), i.e., the middle of the aSLF II, from Y = −21 to Y = −27. The average FA and MD were then extracted from this portion of the aSLF, and correlations between those averages and the aggregated measure of behaviour were computed.

#### 2.4.3 Brain-behaviour correlations

To reduce the number of statistical tests performed while checking for brain-behaviour correlations, we used Discriminant Function Analysis (DFA) to create a single behavioural score. DFA is a statistical technique that predicts group membership based on a set of measures. These measures then act as predictors, with each one of them receiving a weight (similar to a multiple regression coefficient). DFA does not require a minimal number of subjects, unlike logistic regression, the latter of which could not have been performed in the current study for this reason (Tabachnick, Fidell, & Osterlind, 2001). The DFA approach is similar to previous studies that have chosen a single measure to integrate several behavioural measures based on sensitivity to between-group differences (Hyde et al., 2009, Steele et al., 2013).

All behavioural measures that significantly differed between groups were entered into the DFA, and we noted the minimal set of measures that together managed to significantly discriminate between the mathematicians group and the control group. We then employed the standardised canonical coefficient of these selected measures as a weight to aggregate these measures' scores into a single aggregated behavioural score. This aggregated score was then used as a regressor in the VBM analysis seeking brain-behaviour correlations. As an exploratory analysis, we used DFA to compute aggregated scores also for GMD and FA (see Supplementary Methods).

## 3 Results

### 3.1 Behavioural group differences

We observed superior performance for mathematicians versus non-mathematicians in four of our seven numerical tasks. This group difference was also observed in some of the non-numerical tasks, including the verbal reasoning and the logic tasks; and in some of the working memory tasks. There was no group difference in tasks relating to attention and social skills. All behavioural results are summarised in Table 1.

**Table 1:** Summary of the behavioural results, also partly reported elsewhere (Sella, Sader, Lolliot, & Cohen Kadosh, 2016).

Cognitive category	Test	Controls μ ± σ	Mathematicians μ ± σ	Score range	Statistical test
Intelligence	**IQ test (PIQ section)**	114.8 ± 8.4	126.2 ± 6.3	μ = 100, σ = 15	t(36) = 4.71^∗∗∗^
	IQ test (VIQ section)	130.0 ± 5.2	124.1 ± 12.9	μ = 100, σ = 15	n.s. (t(36) = 1.85, *p* = .073)
Working memory	Digit span (forward)	11.2 ± 2.2	11.1 ± 2.7	0 to 16	n.s. (*p* = .95)
	**Digit span (backward)**	7.6 ± 2.4	9.3 ± 2.2	0 to 14	t(36) = 2.26^∗^
	**Letter span (forward)**	9.2 ± 1.5	10.3 ± 1.3	0 to 16	t(36) = 2.16^∗^
Attention	ANT:alerting	18.1 ± 27.3	19.4 ± 18.0	-∞ to ∞	n.s. (*p* = .85)
	ANT:orienting	61.0 ± 34.7	58.1 ± 26.6	-∞ to ∞	n.s. (*p* = .77)
	ANT:executive	100.7 ± 34.8	98.9 ± 35.0	-∞ to ∞	n.s. (*p* = .87)
Mental imagery	**Mental rotation task (MRT)**	10.4 ± 4.3	14.8 ± 4.2	0 to 24	t(36) = 3.20^∗∗∗^
Numerical skills	Number acuity (*w*)	.20 ± .10	.21 ± .13	0 to ∞	n.s. (*p* = .74)
	**Number line (positive numbers)**	62.2 ± 25.8	40.8 ± 14.3	0 to ∞	Mann–Whitney Test: Z = 2.86^∗∗^
	Number line (negative numbers)	59.7 ± 29.7	54.0 ± 23.7	0 to ∞	n.s. (*p* = .52)
	Numerical Stroop	.09 ± .08	.10 ± .04	−1 to 1	n.s. (*p* = .93)
	**Numerical agility**	2.3 ± 2.6	6.5 ± 0.9	0 to 10	Mann–Whitney Test: Z = 4.16^∗∗∗^
	**Numerical strategies**	19.4 ± 7.6	31.7 ± 5.2	0 to 40	t(36) = 3.50^∗∗^
	**Arithmetic task**	13.2 ± 2.1	15.4 ± 2.0	0 to 22	t(36) = 3.29^∗∗^
Logic	**Wason logic task**	.1 ± 0.3	.6 ± 0.5	0 or 1	Fisher's exact *p* = .01, two-tailed
Verbal reasoning	**Verbal reasoning task**	5.5 ± 1.4	5.9 ± 2.7	0 to 10	F(1,35) = 5.51^∗^
Social skills	Emotion recognition task	.94 ± .05	.93 ± .06	0 to 1	n.s. (*p* = .73)
	Gaze task	.93 ± .10	.94 ± .06	0 to 1	n.s. (*p* = .66)
	Face recognition task	48.5 ± 5.0	47.9 ± 2.8	0 to 54	n.s. (*p* = .66)
	Autism spectrum quotient (ASQ)	18.3 ± 7.3	18.1 ± 4.7	0 to 50	n.s. (*p* = .94)
Arithmetic strategies questionnaire	Visuo-spatial	5.21 ± 3.31	7.67 ± 2.74	1 to 10	F(1, 24) = 4.14, *p* = .053
	Inner verbalisation	3.29 ± 2.46	2.08 ± 1.31	1 to 10	n.s. (*p* = .14)
	Outer verbalisation	6.29 ± 2.58	6.00 ± 2.17	1 to 10	n.s. (*p* = .77)
	**Kinaesthetic**	4.93 ± 2.87	2.67 ± 1.50	1 to 10	F(1, 24) = 6.03^∗^

Tests with significant group differences are printed in bold letters.

*p < .05, **p < .01, ***p < .001.

### 3.2 Brain group differences

#### 3.2.1 Grey matter

When contrasting the groups to assess GMD differences at the whole-brain level, no clusters survived the significance level (*p* < .05, corrected for multiple comparisons).

**Fig. 1:**
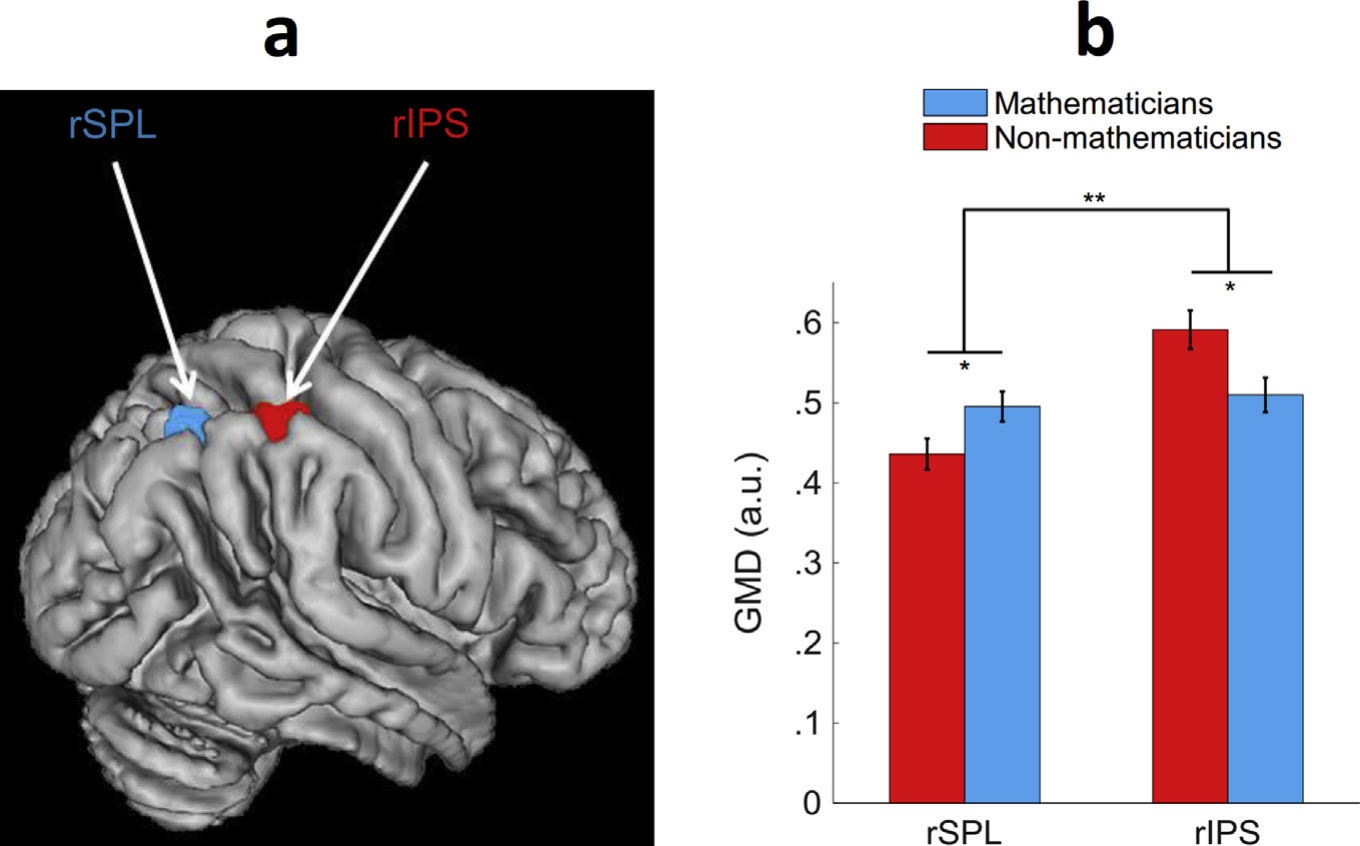
Group differences in GMD, in ROIs of the right hemisphere. **(a)** Higher GMD in mathematicians in a cluster in the right SPL (shown in blue), and higher GMD in non-mathematicians in a cluster in the right IPS (shown in red). (**b**) Mean GMD (expressed in arbitrary units) extracted from each ROI. Error bars represent ±1 standard error of the mean. ROI, region of interest; GMD, grey matter density; rSPL, right superior parietal lobule; rIPS, right intraparietal sulcus. *p < .05, **p < .01.

For the ROI analysis, we tested for group differences in fronto-parietal areas known to be involved in number and arithmetic processing (Arsalidou & Taylor, 2011). Extracted GMD values were submitted to a 3-way mixed-design ANOVA with group (mathematicians, non-mathematicians) as between-subjects factor, and hemisphere (left, right) and defined region (SPL, IPS, and IFG) as within-subject factors. The interaction between region, hemisphere, and group was significant [F(2,72) = 5.59, *p* < .01]. We decomposed the 3-way interaction for each hemisphere. For the right hemisphere, the main effect of group was non-significant (*p* > .4) while the interaction between group and region was significant [F(2,72) = 7.56, *p* < .005]; see Fig. 1. The interaction reflected higher GMD for mathematicians in the right SPL [F(1,36) = 4.80, *p* < .05] but lower GMD for mathematicians in the right IPS [F(1,36) = 6.44, *p* < .05]. For the left hemisphere, the effect of group as well as the group × region interaction were non-significant (all *ps* > .4); we nonetheless decomposed the interaction due to the specific hypotheses we had formed with regard to these regions (Aydin et al., 2007, O'Boyle, 2008), and found lower GMD for mathematicians in the left IFG [F(1,36) = 4.35, *p* < .05].

#### 3.2.2 White matter

TBSS performed at the whole-brain level did not identify any significant voxels that differed in FA across groups (*p* < .05, corrected). There were also no significant group differences in FA or MD for any of the ROIs.

### 3.3 Brain-behaviour correlations

#### 3.3.1 Grey matter

The only behavioural measures that significantly managed to discriminate between the groups in the DFA [F(2, 35) = 26.84, *p* < .0001] were Numerical Strategies and Numerical Agility. Based on each significant predictor's weight sign and associated group comparison (summarised in Table S2 of the Supplementary Materials), a *lower* (more negative) aggregated behavioural score would correspond to *better* performance; in order to make this score more intuitively interpretable, its sign was subsequently inverted (score multiplied by −1); thus, *higher* values corresponded to *better* performance.

**Fig. 2:**
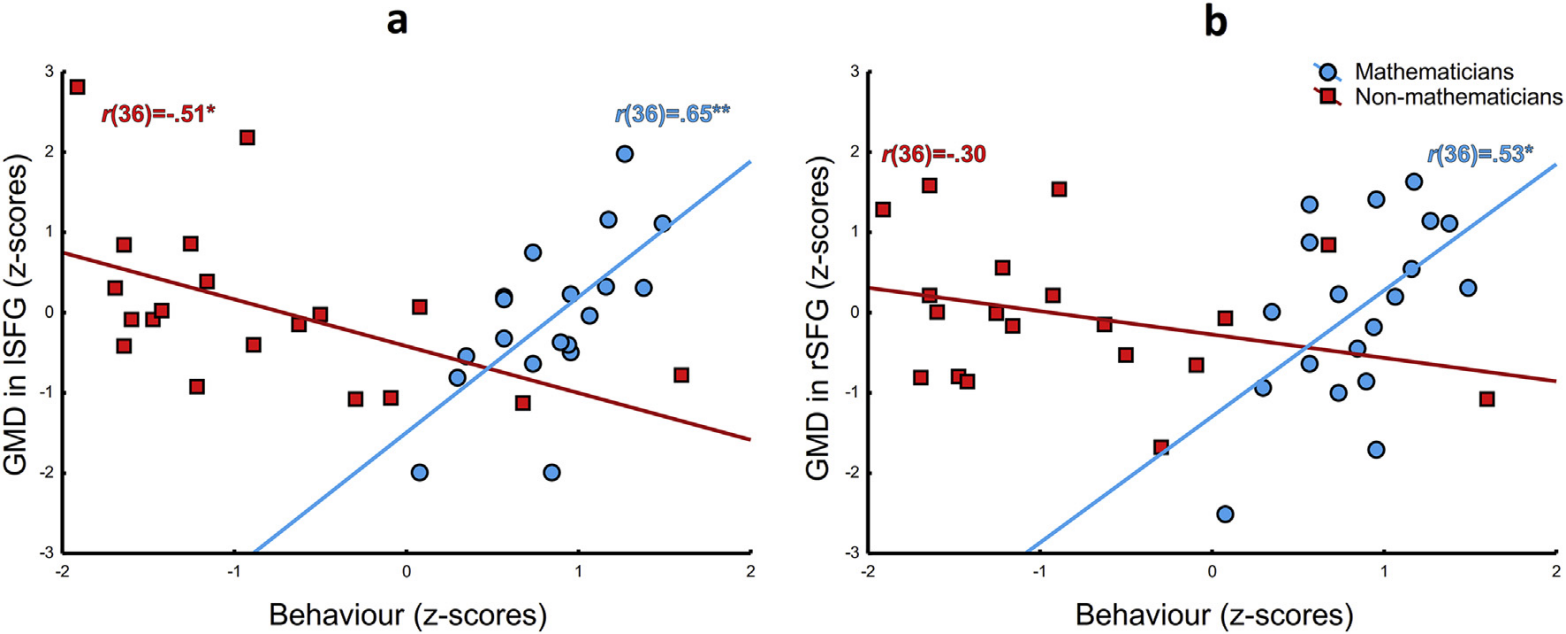
Brain-behaviour correlations, between behavioural score and GMD in the left (**a**) and the right (**b**) SFG. Mathematicians are represented with blue circles, non-mathematicians with red squares. All values are standardised (z-scores). GMD, grey matter density; SFG, superior frontal gyrus. *p < .05, **p < .01.

The VBM whole-brain analysis was run with the aggregated behavioural score as an independent variable. GMD in a 256 mm^3^ cluster in the left superior frontal gyrus (SFG; x = −25, y = −8, z = 68; BA 6), showed a double dissociation, depicted in Fig. 2a: better mathematical performance (as indicated by Numerical Strategies and Numerical Agility) was associated with *lower* GMD in non-mathematicians [*r*(36) = –.51, *p* < .05]; in contrast, better mathematical performance was associated with *higher* GMD in mathematicians [*r*(36) = .65, *p* < .005]. In the contralateral hemisphere, a 91 mm^3^ cluster in the right SFG (x = 24, y = 13, z = 64; BA 8) showed the same correlatory pattern as the left SFG for mathematicians, but not also for non-mathematicians [*r*(36) = .53, *p* < .05 and *r*(36) = –.30, *p* > .1, respectively], as depicted in Fig. 2b.

#### 3.3.2 White matter

Mean FA and mean MD of the white matter in the selected portion of the aSLF, both on the left and on the right, did not correlate significantly with the aggregated behavioural measure (all *p*s > .6).

## 4 Discussion

In order to investigate the neurocognitive bases of mathematical expertise, we compared a group of mathematicians against an academically-matched group of non-mathematicians, on a battery of domain-general and domain-specific tasks, and in terms of brain structure. We found group differences in both the behavioural- and the brain-level measures, as well as in the relationship between them. These results provide new candidates for cortices whose structures might be predictive – or, indeed, a result – of cognitive performance associated with mathematical training. The bidirectionality of the structural group differences observed is in keeping with similar results of unevenly-increased or -decreased metrics obtained within and between studies in the wider literature of brain-structural correlates of expertise. Our results also raise new questions about the relative roles of domain-specific and domain-general ability in creating the higher-level concept of mathematical expertise. Such new questions could inspire future research that will eventually increase our understanding of the neurocognitive mechanisms supporting mathematical ability. While the focus of the present research is on mathematicians *as compared to* non-mathematicians, future studies should examine whether structural differences of the type unveiled here can be linked to the *presence* of other specific types of expertise, as opposed to the *absence* of mathematical expertise.

### 4.1 Behavioural results

Mathematicians were found to display superior performance in a wide range of tasks. These tasks included basic and more complex numerical tasks (e.g., mapping positive numbers on a horizontal line, numerical agility, and numerical strategies). Mathematicians also displayed superior reasoning in the verbal domain; although performance in this task might be attributed to reasoning in a more general sense (Hitch & Baddeley, 1976). Such superior reasoning among mathematicians is consistent with their superior performance on the Wason logic task. There were no group differences in any of the social measures, including the ASQ, which tentatively goes against previous findings (Baron-Cohen et al., 2001, Baron-Cohen et al., 2007), of mathematicians scoring higher on autism-related traits.

Mathematicians demonstrated superior performance in the mental rotation task. This result is in line with previous reports that linked mathematical performance to mental rotation in children, adolescents and adults (Cheng and Mix, 2014, Delgado and Prieto, 2004, Reuhkala, 2001). These differences in mental rotation might be attributed to better visuo-spatial abilities. While we did not examine visuo-spatial abilities directly, in the arithmetic strategies questionnaires mathematicians rated the visuo-spatial modality higher, while non-mathematicians gave higher ratings to kinaesthetic strategies (finger counting). The former confirms older anecdotal reports of mathematicians' increased relative reliance on visuo-spatial thinking (Hadamard, 1949). Other studies, some based on the current behavioural data, do show better visuo-spatial abilities in mathematicians versus non-mathematicians (Hubber et al., 2014, Sella et al., 2016), as well as in abacus experts versus non-experts (Hanakawa, Honda, Okada, Fukuyama, & Shibasaki, 2003).

### 4.2 Brain results

Previous studies have highlighted differences between mathematicians and non-mathematicians in the parietal and the prefrontal cortices (Amalric and Dehaene, 2016, Aydin et al., 2007). Here, for the parietal cortex we found a double dissociation in two closely-located regions; namely, *higher* GMD for mathematicians in the right SPL, but *lower* GMD in the right IPS. This structural double-dissociation mirrors the findings of a previous study on navigational skills, showing higher and lower GMD for taxi drivers in adjacent areas of the same structure, namely the posterior and anterior hippocampus respectively (Maguire et al., 2000). As for the SPL and the IPS, in numerical cognition these have different but complementary roles. The IPS has been linked to sensorimotor integration (among other functions), and in the case of numerical and mathematical cognition to embodiment of numerical information, such as finger counting, and visuo-motor functions (Cohen Kadosh et al., 2011, Di Luca et al., 2006, Kaufmann et al., 2008, Krinzinger et al., 2011). In contrast, the SPL has been linked to visuo-spatial processing of numerical information (Dehaene et al., 2003, Knops et al., 2009). That this finding might indicate more abstract processing in mathematicians versus more embodied processing in non-mathematicians is an attractive possibility, supported to some degree by our results from the arithmetic strategies questionnaire, and that will require further research.

In addition, we observed lower GMD in the left IFG. This result is based on a non-significant interaction, which we decomposed based on a previous study that had observed differences in the IFG between mathematicians and non-mathematicians (Aydin et al., 2007), and as such should be taken with caution. The IFG is involved in different cognitive functions, including calculation (for review, see Arsalidou and Taylor, 2011, Moeller et al., 2015), and it is difficult to assign the reason for such differences, especially given the cross-sectional design of our study. One possibility is that the GMD difference in this structure might relate to differences in strategies during arithmetic, as indicated by the current study and previous ones (e.g., Dowker, 1992). Another possibility could be that it instead relates to the group difference that we observed in fluid intelligence, as measured by the performance IQ. Indeed, when we included performance IQ as a covariate in our 3-way ANOVA, the group difference in GMD lost its significance in the case of the left IFG [F(1,35) = 1.26, *p* = .27], but maintained or trended towards it in the case of the right SPL [F(1,35) = 4.97, *p* < .05] and the right IPS [F(1,35) = 3.85, *p* = .058].

We found lower right IPS GMD for mathematicians versus non-mathematicians. This finding paradoxically mirrors what was found previously in some studies on dyscalculia: *lower* GMD is found in the right IPS as compared to healthy controls (Cappelletti and Price, 2014, Molko et al., 2003, Rotzer et al., 2008, Rykhlevskaia et al., 2009). Future studies that will include those with low, average, and exceptional mathematical abilities could examine whether at least in the case of right IPS, local GMD might have a non-linear relationship with mathematical ability. Previous reports linking expertise in various cognitive domains to GMD in relevant regions have found – for the expert group with respect to relevant controls – either *higher* GMD (Aydin et al., 2007, Maguire et al., 2000, Scholz et al., 2009, Sluming et al., 2002), *lower* GMD (Hänggi, Koeneke, Bezzola, & Jäncke, 2010), or a mixture of the two across regions (Han et al., 2012, James et al., 2013).

Our GMD results included differences between mathematicians and non-mathematicians in the right IPS and right SPL, but not in the contralateral hemisphere. This might suggest that these differences are rooted in visuo-spatial abilities that are more right-lateralised, especially with respect to the parietal cortex (de Hevia et al., 2008, Miller et al., 2018). While this suggestion would need further validation, the current results do also suggest a tendency of higher usage of visuo-spatial strategies to solve arithmetic problems by mathematicians. Moreover, Sella et al. (2016) demonstrated in the current sample that the relation between basic, even specific, numerical skills and advanced mathematical achievement can be artefactual and explained by visuo-spatial processing.

It is important to note the discrepancy between our findings and those of Aydin et al. (2007). That study found higher GMD in mathematicians in the left inferior frontal and in the bilateral inferior parietal lobules. In contrast, for mathematicians we observed lower GMD in the left IFG and right IPS, no differences in the left IPS, and higher GMD in the right SPL. These differences are difficult to explain and would require further studies in order to test the robustness of the findings in both studies, as well as the contribution of differences in the design, such as the age of the participants (on average 10 years older in Aydin et al.'s study), and, inseparably, their academic seniority (PhD candidates and postdocs in the current study *vs* approx. 14 years after university in Aydin et al.'s study).

We found that the SFG in both hemispheres is positively correlated with the DFA-aggregated behavioural score (based on Numerical Strategies and Numerical Agility) in mathematicians. In contrast, in non-mathematicians only the left SFG showed a significant – and negative – correlation. The SFG has been shown previously to play a role in arithmetic and non-arithmetic processing (Arsalidou and Taylor, 2011, Buchsbaum et al., 2005). While we did not expect the dissociation in the SFG a priori, the similar finding in both hemispheres, might suggest that it is less likely to be due to a type I error. It is difficult to be certain on the role of the SFG in the current case, although one possible explanation is its role in mental flexibility. This idea is in line with the finding that mathematicians are more likely to shift between different strategies when solving the problems involved in the Numerical Strategies and Numerical Agility tasks, rather than using a single strategy as in the case of non-mathematicians (Dowker, 1992). Further efforts would be needed to examine this possibility.

We did not find differences between the groups in terms of white matter's FA. This comes in contrast with the results of previous studies that have examined numerical cognition using DTI. For instance, Matejko et al. (2013) found higher FA in the left SLF, superior corona radiata (SCR) and the cortico-spinal tract to be associated with better performance in an arithmetic test. In addition, Tsang et al. (2009) found that, in children, mean FA in the aSLF correlates with behavioural performance. While a null result can be attributed to different factors, potential explanations for ours could relate to the different populations and the different tasks used to examine the brain-behaviour correlation across these studies. For instance, Tsang et al. (2009) studied children while the current study used adults; and the correlation they report between the aSLF FA values and arithmetic skills was based on a mental as opposed to a written arithmetic task as used in the current study.

## 5 Conclusions

Similar to previous studies, we found performance differences between mathematicians and non-mathematicians in both domain-general and domain-specific cognitive processes. The novelty in our study are the structural brain differences between mathematicians and non-mathematicians, including the double dissociations between the GMD in the right SPL and in the right IPS; and between behaviour and GMD in the left and right SFG. At the same time, we did not observe differences in measures of white matter; such null results are important to document for future studies, in order to assess the sensitivity of macrostructural white matter measures in high performing adults (see, e.g., Zatorre, Fields, & Johansen-Berg, 2012).

In the current study, we provided a first step towards exploring the neurocognitive differences between mathematicians, independently of their field, and non-mathematicians. While such a comparison is likely to mask more subtle differences that exist between the various mathematical fields (e.g., geometry *vs* number theory), it does, nonetheless, allow us to infer general differences between mathematicians and non-mathematicians. How and at what developmental stages such differences arise; how lower or higher GMD is related to mathematical expertise, strategic usage, and mathematical education; and how these influence and are influenced by domain-specific and domain-general cognitive processes, are all important questions that arise from the current results, and that we hope will motivate future studies.

## Competing financial interests

The authors declare that the research was conducted in the absence of any commercial or ﬁnancial relationships that could be construed as a potential conﬂict of interest.

## Footnotes

1 We refer to this group as the non-mathematicians rather than as a control group, in recognition of the fact that any selection of an “amathematical” control group is necessarily arbitrary with respect to the expertise that characterises it. Thus, one might – perhaps more accurately – refer to this as the “contrast group” (Wilkinson, 1999).
