# Supplementary material for: The brain-structural correlates of mathematical expertise
Source: Cortex. 2019 May;114:140–50. doi: 10.1016/j.cortex.2018.10.009 (PMC6996130; doi:10.1016/j.cortex.2018.10.009)
Supplement: Multimedia component 1 [file mmc1.docx]

# Supplementary Methods

## Behavioural tasks

Table S1: Summary of the battery of cognitive tests used.

| Cognitive category | Test name | Reference | Representative task screenshot |
| --- | --- | --- | --- |
| Intelligence | IQ test  Wechsler Abbreviated Scale of Intelligence (WASI) | (Wechsler, 1999) | 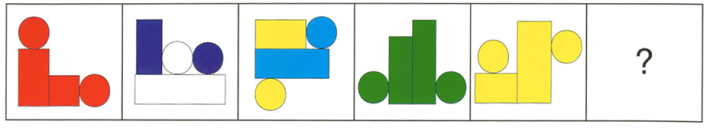 |
| Working memory | Digit span (backward&forward) | (Wechsler, 1997) | 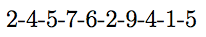 |
|  | Letter span (forward) |  | 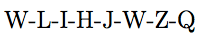 |
| Attention | Attention networks task (ANT) | (Fan, McCandliss, Sommer, Raz, & Posner, 2002) | 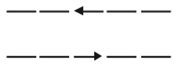 |
| Mental imagery | Mental rotation task (MRT) | (Peters et al., 1995) | 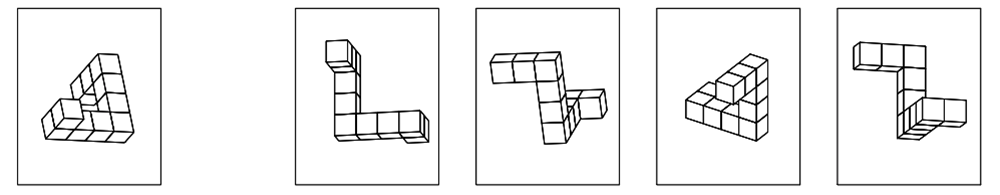 |
| Numerical skills | Number acuity | (based on Piazza, Izard, Pinel, Le Bihan, & Dehaene, 2004) | 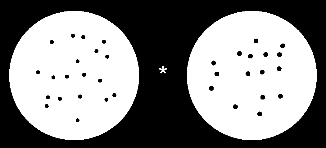 |
|  | Number line task | (based on Siegler & Opfer, 2003) | **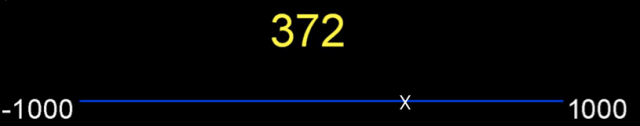** |
|  | Numerical Stroop | (Henik & Tzelgov, 1982) | 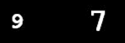 |
|  | Numerical agility | (Sella, Sader, Lolliot, & Cohen Kadosh, 2016) | 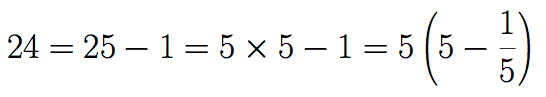 |
|  | Numerical strategies | (based on Levine, 1982) | 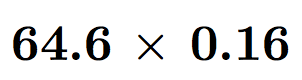 |
|  | Arithmetic task | (based on Wechsler, 1997) | 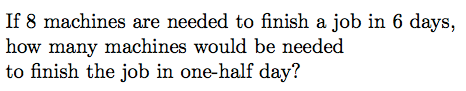 |
| Logic | Wason logic task | (Wason, 1966) | 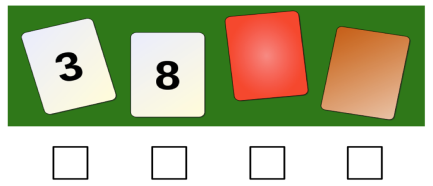 |
| Verbal reasoning | Verbal reasoning task | (Association of American Medical Colleges, 2007) | 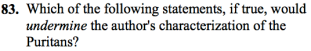 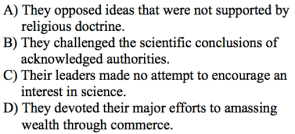 |
| Social skills | Emotion recognition task | (Cohen Kadosh, Henson, Cohen Kadosh, Johnson, & Dick, 2010) | 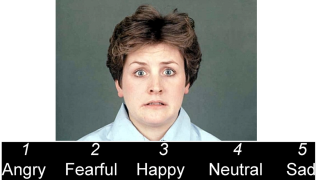 |
|  | Gaze task | (Cohen Kadosh et al., 2010) | **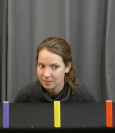** |
|  | Face recognition task | (Benton, Van Allen, Hamser, & Levin, 1978) | 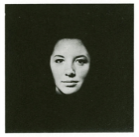 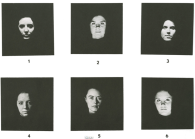 |
|  | Autism spectrum quotient (ASQ) | (Baron-Cohen, Wheelwright, Skinner, Martin, & Clubley, 2001) | 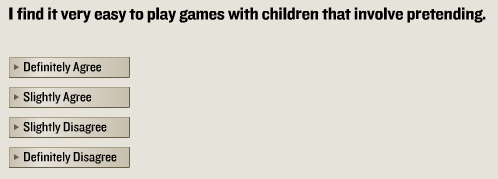 |

## Aggregated scores for GMD and FA

As an exploratory analysis, we used DFA to compute aggregated scores also for GMD and FA measures. As with the computation of the behavioural score, for GMD we started by entering into the DFA those scores (ROIs) that were significantly different between groups, namely the right IPS, right SPL and left IFG. FA from the same ROIs was entered into the DFA for the computation of the FA score, despite these not having significantly differed between the groups.

# Supplementary Results

## Brain results

As mentioned in the main paper, the behavioural measures that significantly managed to discriminate between the groups (F(2, 35)=26.84, p<.0001) were Numerical Strategies and Numerical Agility. For GMD, all three ROIs entered into the DFA were also significant predictors (F(3,34)=6.74, *p*<.005). For FA, it was only the ROIs underlying the left IFG and right IPS that were significant (F(2,35)=3.44, *p*<.05).

Based on each significant predictor's weight sign and associated group comparison (summarised in Table S3 below), it can be inferred that a *lower* (more negative) aggregated behavioural score would correspond to *better* performance; in order to make this score more intuitively interpretable, it was subsequently inverted (multiplied by -1); thus, *higher* values corresponded to *better* performance. On the other hand, a higher aggregated GMD score meant greater GMD values in right SPL and smaller GMD values in right IPS and left IFG; and a higher aggregated FA score meant greater FA values in right IPS and smaller FA values in left IFG. We therefore avoid the interpretation of the GMD and FA scores in terms of there being higher or lower GMD and FA in the whole of the network of interest. There was a significant correlation between the aggregated scores for behaviour and GMD (r=.41, *p*<.05), between behaviour and FA (r=-.36, *p*<.05), and between GMD and FA (r=.34, *p*<.05).

Table S2: Summary of significant DFA predictors in each of the three measurement categories. The second column indicates, in brackets, the group difference in the predictor, at the descriptive and inferential level (indicated by asterisks). The third column indicates the predictor's weight and significance within the DFA analysis itself. * *p*<.05, ** *p*<.01, *** *p*<.001.

| Aggregated score | Predictor [group comparison] | Predictor weight [significance of Wilks' Lambda] |
| --- | --- | --- |
| Behaviour | Numerical Strategies [M>C *] | -0.49 * |
|  | Numerical Agility [M>C *] | -0.69 *** |
| GMD | rSPL [M>C *] | 0.75 ** |
|  | rIPS [C>M *] | -0.65 * |
|  | lIFG [C>M *] | -0.63 * |
| FA | rIPS [C>M] | 1.05 * |
|  | lIFG [M>C] | -1.17 * |

# Supplementary references

Baron-Cohen, S., Wheelwright, S., Skinner, R., Martin, J., & Clubley, E. (2001). The Autism-Spectrum Quotient (AQ): Evidence from Asperger Syndrome/High-Functioning Autism, Malesand Females, Scientists and Mathematicians. *Journal of Autism and Developmental Disorders*, *31*(1), 5–17.

Benton, A., Van Allen, M., Hamser, K., & Levin, H. (1978). *Test of Facial Recognition Manual*.

Cohen Kadosh, K., Henson, R. N. A., Cohen Kadosh, R., Johnson, M. H., & Dick, F. (2010). Task-dependent Activation of Face-sensitive Cortex: An fMRI Adaptation Study. *Journal of Cognitive Neuroscience*, *22*(5), 903–917. https://doi.org/10.1162/jocn.2009.21224

Fan, J., McCandliss, B. D., Sommer, T., Raz, A., & Posner, M. I. (2002). Testing the Efficiency and Independence of Attentional Networks. *Journal of Cognitive Neuroscience*, *14*(3), 340–347. https://doi.org/10.1162/089892902317361886

Henik, A., & Tzelgov, J. (1982). Is three greater than five: The relation between physical and semantic size in comparison tasks. *Memory & Cognition*, *10*(4), 389–395.

Levine, D. R. (1982). Strategy Use and Estimation Ability of College Students. *Journal for Research in Mathematics Education*, *13*(5), 350–359. https://doi.org/10.2307/749010

Peters, M., Laeng, B., Latham, K., Jackson, M., Zaiyouna, R., & Richardson, C. (1995). A Redrawn Vandenberg and Kuse Mental Rotations Test - Different Versions and Factors That Affect Performance. *Brain and Cognition*, *28*(1), 39–58. https://doi.org/10.1006/brcg.1995.1032

Piazza, M., Izard, V., Pinel, P., Le Bihan, D., & Dehaene, S. (2004). Tuning curves for approximate numerosity in the human intraparietal sulcus. *Neuron*, *44*(3), 547–555.

Sella, F., Sader, E., Lolliot, S., & Cohen Kadosh, R. (2016). Basic and Advanced Numerical Performances Relate to Mathematical Expertise but Are Fully Mediated by Visuospatial Skills. Retrieved from http://psycnet.apa.org/psycinfo/2016-10025-001/

Siegler, R. S., & Opfer, J. E. (2003). The Development of Numerical Estimation: Evidence for Multiple Representations of Numerical Quantity. *Psychological Science*, *14*(3), 237–250. https://doi.org/10.1111/1467-9280.02438

Wason, P. C. (1966). Reasoning. *New Horizons in Psychology*, *1*, 135–151.

Wechsler, D. (1997). *WAIS-III/WMS-III technical manual*. San Antonio, TX: The Psychological Corporation.

Wechsler, D. (1999). Manual for the Wechsler abbreviated intelligence scale (WASI). *San Antonio, Tex: The Psychological Corporation*.
